# Injection of Adipose-Derived Mesenchymal Stem/Stromal Cells Suppresses Muscle Atrophy Markers and Adipogenic Markers in a Rat Fatty Muscle Degeneration Model

**DOI:** 10.3390/cimb46080467

**Published:** 2024-07-24

**Authors:** Sai Koung Ngeun, Miki Shimizu, Masahiro Kaneda

**Affiliations:** 1 Department of Veterinary Diagnostic Imaging, Faculty of Agriculture, Tokyo University of Agriculture and Technology, 3-5-8 Saiwai-cho, Fuchu 183-8509, Tokyo, Japan; s212892q@st.go.tuat.ac.jp; 2 Department of Veterinary Anatomy, Faculty of Agriculture, Tokyo University of Agriculture and Technology, 3-5-8 Saiwai-cho, Fuchu 183-8509, Tokyo, Japan; kanedam@cc.tuat.ac.jp

**Keywords:** histopathology, gene expression, ADP MSCs, myogenic differentiation potential, immunomodulatory

## Abstract

Fatty muscle degeneration and muscle atrophy have not been successfully treated due to their irreversible pathology. This study evaluated the efficacy of rat adipose-derived mesenchymal stem/stromal cells (ADP MSCs) in treating fatty muscle degeneration (FD). A total of 36 rats were divided into three groups: the control (C) group (*n* = 12); FD model group, generated by sciatic nerve crushing (*n* = 12); and the group receiving ADP MSC treatment for FD (FD+MSCs) (*n* = 12). In Group FD+MSCs, ADP MSCs were injected locally into the gastrocnemius muscle one week after the FD model was created (Day 8). On Day 22 (*n* = 18) and Day 43 (*n* = 18), muscle morphology, histopathology, and molecular analyses (inflammation, muscle atrophy, adipocytes, and muscle differentiation markers) were performed. In Group FD+MSCs, the formation of immature myofibers was observed on Day 22, and mitigation of fatty degeneration and muscle atrophy progression was evident on Day 43. Gene expression of muscle atrophy markers (*FBXO32*, *TRIM63*, and *FOXO1*) and adipogenic markers (*ADIPOQ*, *PPARG*, *FABP4*, and *PDGFRA*) was lower in Group FD+MSCs than Group FD on Day 43. ADP MSCs induce anti-inflammatory effects, inhibit fat accumulation, and promote muscle regeneration, highlighting their potential as promising therapy for FD and atrophy.

## 1. Introduction

The structural composition of muscle plays a pivotal role in determining its strength and functionality [1]. Furthermore, any decrease in muscle quality may lead to a decline in functional activities [2]. Therefore, maintaining muscle mass is crucial due to its role in regulating the body’s metabolic systems and supporting daily activities that demand mobility, strength, and power [3]. A lack of physical activity leads to increased levels of intermuscular fat deposition and reduced muscle mass and strength [4] and elevates the risk of fracture [5]. Fat accumulation, along with muscle degeneration and atrophy, resulting in muscle dysfunction, is termed fatty muscle degeneration [6]. This condition is prevalent in various degenerative muscular diseases, including chronic muscle and tendon injury, inherited neuromuscular disorders, aging, insulin resistance, and type 2 diabetes [7,8]. Due to its secondary nature and the absence of obvious clinical signs, it can be easily overlooked during the early stages, leading to limited awareness of the condition. Despite successful repairs of tendon ruptures and nerve injuries, muscle degeneration or fatty infiltration remains irreversible in certain cases [9], emphasizing the necessity for therapeutic interventions.

Fibro-adipogenic progenitors (FAPs) are a population of mesenchymal cells found in the interstitial spaces of muscles, which support satellite cell differentiation during tissue regeneration [10]. FAPs reside in the interstitial space of skeletal muscle, contributing to either muscle regeneration in healthy tissue or fibrosis and fat deposition in pathological conditions [11]. In chronically degenerated muscle, there is impaired regulatory cross-talk between inflammatory cytokines, such as transforming growth factor-beta (TGF-β), tumor necrosis factor-alpha (TNF-α), and FAPs, resulting in muscle fibrosis [10]. The regenerative capacity of skeletal muscle relies on satellite cells, and retaining satellite cells may be achieved by suppressing the accumulation of intramuscular fat [12]. Intermuscular fat is considered to be an ectopic fat depot, similar to visceral adipose tissue found in the abdomen [1]. It is capable of releasing proinflammatory cytokines that induce local inflammation and fatty muscle degeneration [13,14]. Preventing intramuscular fat buildup has the potential to preserve satellite cells and reduce inflammation, thereby preventing fatty muscle degeneration. Currently, there are no successful treatments for fatty muscle degenerative diseases.

Mesenchymal stem/stromal cells (MSCs) are multipotent stem cells. They possess myogenic differentiation abilities [15] and immunomodulatory properties, as evidenced by the expression of insulin-like growth factor (*IGF*), transforming growth factor beta 1 (*TGFB1*), and interleukin 6 (*IL6*) [16]. Additionally, their clinical application is more suitable due to fewer legal controversies and lower tumorigenicity [17]. Adipose-derived MSCs (ADP MSCs) are easier to isolate and proliferate more rapidly than bone marrow-derived MSCs. Therefore, ADP MSCs are a promising alternative for potential use in musculoskeletal diseases [18]. The immunomodulatory properties enable MSCs to alleviate inflammation when the immune system is overactive and enhance inflammation when the immune system is inactive [19]. Therapeutically, the transplantation of rat bone marrow-derived MSCs into muscle injuries has shown promise in enhancing muscle regeneration by enhancing myoblast proliferation [20,21]. Takegaki et al. [22] have also reported that the injection of MSCs increases the upregulation of genes associated with satellite cell and muscle protein synthesis. Additionally, MSCs exhibit the capacity to secrete growth factors such as fibroblast growth factor (FGF), hepatocyte growth factor (HGF), and IGF1, promoting myoblast migration, multiplication, differentiation, and ultimately facilitating muscle regeneration [23,24,25].

Research on the therapeutic effects of MSCs on fatty infiltration and atrophy of muscle is limited. Flück et al. [26] report that bone marrow-derived MSC transplantation prevents fatty degeneration of the detached rotator cuff muscle in a sheep model after tendon repair. Extracellular vesicles from human umbilical cord stem cells prevent fatty degeneration of the rotator cuff muscle after tendon repair in rabbits [27]. Fatty muscle infiltration and atrophy were less intense following the transplantation of stromal stem cells in the tenotomized infraspinatus muscle of sheep [28]. However, the therapeutic effect of rat ADP MSCs in relation to the molecular markers and physiological mechanisms underlying muscle regeneration, such as inflammatory response, is limited [29].

Therefore, this study aimed to evaluate the therapeutic potential of rat ADP MSCs in fatty muscle degeneration, using histopathological and molecular analysis of fatty degeneration due to sciatic nerve crush injury in a rat model. We hypothesized that rat ADP MSCs could suppress inflammation and fat accumulation, and facilitate myogenic differentiation through satellite cell activation, thereby reversing fatty muscle degeneration and atrophy.

## 2. Materials and Methods

### 2.1. Experimental Animals and Experimental Design

**Figure 1:**
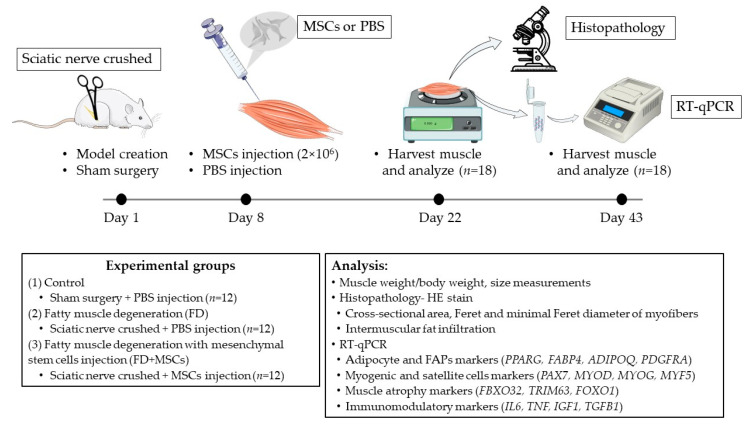
Experimental design. MSCs, mesenchymal stem/stromal cells; PBS, phosphate-buffered saline; FAPs, fibro-adipogenic progenitors; HE stain, hematoxylin, and eosin stain (illustrations source: https://bioicons.com/ (accessed on 15 January 2024)).

A total of 36 male Wistar rats, aged 10 weeks, were divided into 3 groups: Control (C, *n* = 12), fatty muscle degeneration (FD, *n* = 12), and fatty muscle degeneration with MSCs injection (FD+MSCs, *n* = 12). The rats were housed in cages at room temperature ranging from 18 to 23 °C, with a day/night cycle of 12 h. They were provided with ad libitum access to laboratory animal dry food and water. Fatty muscle degeneration was induced by crushing the sciatic nerve on the left side to induce fatty degeneration of the left gastrocnemius muscle, while the control group (C) underwent a sham surgery without crushing the sciatic nerve. Six rats from each group were analyzed at 3 and 6 weeks (Day 22 and Day 43, respectively) after the model was established. The left gastrocnemius muscles were harvested following humane euthanasia, achieved by continuous exposure to isoflurane until cessation of breathing. If necessary, death was confirmed by percutaneous cardiac puncture to confirm the absence of a heartbeat. All animal experiments were conducted under the approval and guidance of the Animal Care and Use Committee of the Tokyo University of Agriculture and Technology (approval numbers: R05-145, approved on 23 May 2023). Figure 1 provides an overview of the experimental design.

### 2.2. Establishment of Fatty Muscle Degeneration Model by Sciatic Nerve Injury

**Figure 2:**
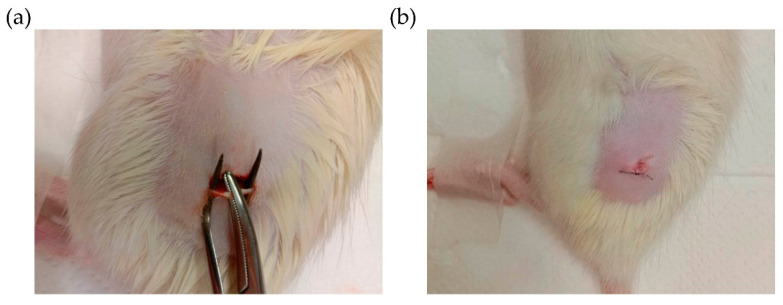
Fatty muscle degeneration model. (**a**) Creation of the model in the gastrocnemius muscle via sciatic nerve crushing. (**b**) Closure of the surgical site after model creation.

All surgical interventions were conducted under disinfection conditions using inhalation anesthesia: isoflurane at 2–3% with an oxygen flow ranging from 1 to 2 L per minute, complemented by subcutaneous injection of bupivacaine (1 mg/kg) as local anesthesia. The procedure involved making an incision in the skin of the rats’ left hind limbs, followed by the separation of the vastus lateralis and biceps femoris muscles to expose the sciatic nerve. The sciatic nerve injury model was established by clamping the nerve using hemostatic forceps for one minute, positioned 5 mm proximal to the bifurcation point of the peroneal and tibial nerves (Figure 2a) [30].

The skin incision was closed with a 4-0 nylon suture (Figure 2b). Enrofloxacin at 10 mg/kg/day and meloxicam at 1 mg/kg/day were administered subcutaneously to prevent post-surgical infections and pain [15].

### 2.3. Injection of Mesenchymal Stem Cells

In Group FD+MSCs, adipose-derived MSCs (ADP MSCs) obtained in our previous experiment [31] were injected into the left gastrocnemius muscle. We confirmed that the isolated cells were MSCs according to the criteria set by the International Society for Cellular Therapy [32]. For the isolation of ADP MSCs, adipose tissues were collected from the subcutaneous layer around the scapula region of rats. The tissues were minced and digested with 0.1% collagenase and then centrifuged to obtain cell pellets. These cell pellets were resuspended in a culture medium composed of Dulbecco’s Modified Eagle Medium (catalog no. 043-30085, FUJIFILM Wako Pure Chemical Corporation, Osaka, Japan), 20% fetal bovine serum (catalog no. CCP-FBS-BR-500, COSMO BIO, Tokyo, Japan), 1% non-essential amino acids (catalog no. 139-15651, FUJIFILM Wako Pure Chemical Corporation, Osaka, Japan), and 1% Penicillin/Streptomycin (catalog no. 161-23181, FUJIFILM Wako Pure Chemical Corporation, Osaka, Japan) and cultured at 37 °C in a humidified environment with 5% CO_2_. Cells were cultured until passage 4. For this research, ADP MSCs were detached from the culture dish using trypsin. The reaction was stopped by adding culture medium, followed by centrifugation at 1500 rpm for 2 min to obtain a cell pellet. Subsequently, the cells were counted, totaling 2 × 10^6^ ADP MSCs, which were then resuspended in 0.2 mL of phosphate-buffered saline and locally injected into the left gastrocnemius muscle one week after establishing the model [22]. In Groups C and FD, 0.2 mL of phosphate-buffered saline was administered into the left gastrocnemius muscle.

### 2.4. Gross and Histopathological Examination

The left gastrocnemius muscles were harvested and their wet weight and size (length × width × height) were measured using a digital scale and vernier caliper to confirm the atrophic changes of the muscles. For the observation of muscle fiber atrophy and fatty muscle degeneration, Hematoxylin and Eosin (HE) staining was performed.

For the histological slide preparation, the proximal half of the muscles was fixed in 10% formalin at room temperature for 24 h. After fixation, the tissue was gently dehydrated by immersion in methanol. Subsequently, the dehydrating agent was cleared by incubation in xylene before being embedded in paraffin. The paraffin-embedded specimens were cut with a microtome to a thickness of 3 µm, mounted on glass slides, and air-dried for 2 h. The slides were then deparaffinized in xylene, rehydrated with ethanol, washed in running water, and stained with HE. HE-staining was performed using Carrazi’s Hematoxylin (catalog no: 3002-2, MUTO PURE CHEMICALS Co., Ltd., Tokyo, Japan) and 1% Eosin Y solution (catalog no: 051-06515, FUJIFILM Wako Pure Chemical Corporation, Osaka, Japan).

The histological slides were used to assess the cross-sectional area, Feret diameter, and minimal Feret diameter of muscle fibers, adipocyte infiltration, and histopathological architecture differences. After defining the region of interest, 400 muscle fibers per sample were continuously measured using ImageJ software version 1.53k (https://imagej.nih.gov/ij/download.html (accessed on 8 January 2022)) and employed for statistical analysis. All slides were observed using a digital imaging microscope (BZ-9000, KEYENCE, Osaka, Japan).

### 2.5. Reverse Transcription-Quantitative Polymerase Chain Reaction (RT-qPCR)

For gene expression analysis, the distal half of the muscles was harvested and stored in RNAlater^®^ (catalog no. R0901-500ML, Sigma-Aldrich, St. Louis, MO, USA) at −80 °C, until RNA isolation. The TRIzol method was utilized for total RNA extraction. The muscle tissues were homogenized in 1 mL of TRIzol (catalog no. 15596026, Thermo Fisher Scientific Inc., Life Technologies Corporation, Carlsbad, CA, USA). Further homogenization was conducted using the QIAshredder by centrifuging samples at 4 °C for 15 min at 12,000× *g* (catalog no. 79654, QIAGEN, Hilden, Germany). The homogenized lysate was then transferred to a 2 mL microcentrifuge tube and incubated with 0.2 mL of chloroform (catalog no. 038-02606, FUJIFILM Wako Pure Chemical Corporation, Osaka, Japan) at room temperature for 5 min. The colorless upper aqueous phase was transferred to a new 2 mL microcentrifuge tube, and the extraction process was repeated by adding 0.5 mL of chloroform. After centrifugation at 4 °C for 15 min at 12,000× *g*, the colorless upper aqueous phase was transferred to a new tube and incubated with 0.5 mL of isopropanol (catalog no. 29113-95, Nacalai Tesque, Inc., Kyoto, Japan) at room temperature for 5 min. Following centrifugation at 4 °C for 15 min at 12,000× *g*, the RNA pellet was collected. Contaminants were removed by washing with 1 mL of room temperature RNase-free 70% ethanol (catalog no: 057-00456, FUJIFILM Wako Pure Chemical Corporation, Osaka, Japan) and centrifugation at 12,000× *g* for 5 min at 4 °C. The RNA samples were then air-dried for 10–30 min and diluted in 50 µL of DEPC-treated water (catalog no. 36415-54, Nacalai Tesque, Inc., Kyoto, Japan) for each sample. The isolated RNA underwent treatment with the TURBO DNAfree™ Kit (catalog no. AM1907, Thermo Fisher Scientific Inc., New York, NY, USA) to eliminate DNA contamination. Assessment of RNA quantity was conducted utilizing a NanoDrop^TM^ Lite spectrophotometer (catalog no. ND-LITE-PR, Thermo Fisher Scientific Inc., Wilmington, DE, USA). Subsequently, the first-strand cDNA was synthesized using the ReverTra Ace^®^ qPCR RT Master Mix (code no. FSQ-201, TOYOBO, Osaka, Japan), according to the manufacturer’s instructions.

The expression levels were quantified in a relative manner by utilizing the Applied Biosystems StepOnePlus™ Real-Time PCR System (Thermo Fisher Scientific, Waltham, MA, USA). The RT-qPCR setup included 2 µL cDNA, 0.5 µL of each forward and reverse primer (10 µmol/L), 10 µL THUNDERBIRD^®^ Next SYBR^®^ qPCR Mix (code no. QPX-201, TOYOBO, Osaka, Japan), and 7 µL dH_2_O. The thermal cycling conditions for qPCR were 95 °C for 30 s, followed by 40 amplification cycles. Each cycle comprised of a denaturation step at 95 °C for 5 s and an annealing/extension step at 60 °C for 10 s. The relative quantification of gene expression was determined using the 2^−△△CT^ method and normalized to *GAPDH* as a reference gene.

Regarding the molecular confirmation of fatty muscle degeneration and the therapeutic effect of ADP MSCs, the expression levels of muscle atrophy markers (F-box protein 32 (*FBXO32*), tripartite motif containing 63 (*TRIM63*), and forkhead box O1 (*FOXO1*)); adipocyte markers (peroxisome proliferator-activated receptor-gamma (*PPARG*), fatty acid-binding protein 4 (*FABP4*), and adiponectin (*ADIPOQ*)); FAPs marker (platelet-derived growth factor receptor alpha, *PDGFRA*); myogenic and satellite cell markers (paired box 7 (*PAX7*), myogenic differentiation protein (*MYOD*), myogenin (*MYOG*), and myogenic factor 5 (*MYF5*)); and immunomodulatory markers *IL6*, *TNF*, *IGF1*, and *TGFB1* were validated at the mRNA level through RT-qPCR. The relative expression level of glyceraldehyde-3-phosphate dehydrogenase (*GAPDH*) among Group C, FD, and FD+MSCs was statistically analyzed and presented as the median with an interquartile range of (*n* = 6). The specific primers used for the RT-qPCR reactions are detailed in Table 1.

### 2.6. Statistical Analysis

Statistical analysis of all data was conducted using GraphPad Prism software version 8.4 (GraphPad Software, Inc., La Jolla, CA, USA). The data are presented as the median with interquartile range, and between-group comparisons were conducted using the Kruskal–Wallis test, followed by Dunn’s multiple comparison test. A significance level of *p* < 0.05 was considered statistically significant.

## 3. Results

### 3.1. Gross and Histopathological Examination

**Figure 3:**
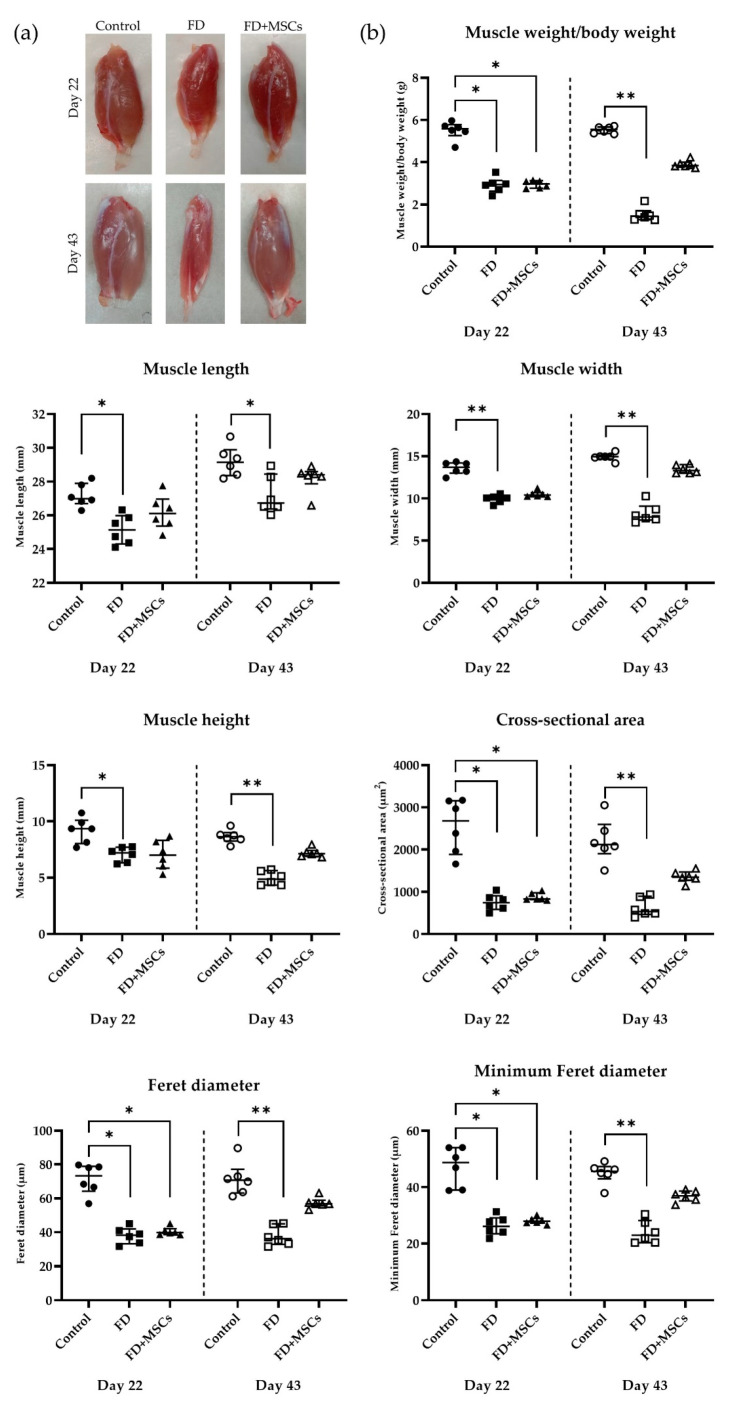
Gross examination and measurements of the gastrocnemius muscle on Day 22 and Day 43. (**a**) Representative gross examination images and (**b**) measurements of the muscle weight/body weight, length, width, height, cross-sectional area, Feret, and minimum Feret diameter. The Kruskal–Wallis test and Dunn’s multiple comparison test were utilized for data analysis and shown as the median (wide horizontal line) with interquartile range (narrow horizontal line) (*n* = 6) (* *p* < 0.05, ** *p* < 0.001). Group C on Day 22, filled circle; Group FD on Day 22, filled rectangle; Group FD+MSCs on Day 22, filled triangle; Group C on Day 43, unfilled circle; Group FD on Day 43, unfilled rectangle; Group FD+MSCs on Day 43, unfilled triangle; C, control; FD, fatty muscle degeneration; FD+MSCs, fatty muscle degeneration with mesenchymal stem cell injection.

The gross examination of the harvested gastrocnemius muscles is shown in Figure 3a. Measurements including muscle weight/body weight, length, width, and height of the rat’s gastrocnemius muscles on Day 22 and Day 43 are shown in Figure 3b. On Day 22, the gastrocnemius muscle weight/body weight of Group FD and Group FD+MSCs were lower than Group C (*p* = 0.0062 and *p* = 0.0175, respectively). On Day 43, the gastrocnemius muscle weight/body weight of Group FD continued to decrease compared to Group C (*p* = 0.0003), whereas Group FD+MSCs did not show a difference compared to Group C. On Day 22, the length, width, and height of the gastrocnemius muscle in Group FD were lower than in Group C (*p* = 0.0062, *p* = 0.0009, and *p* = 0.0386, respectively). On Day 43, the length, width, and height of the gastrocnemius muscle in Group FD were still lower than in Group C (*p* = 0.0283, *p* = 0.0003, and *p* = 0.0004, respectively).

**Figure 4:**
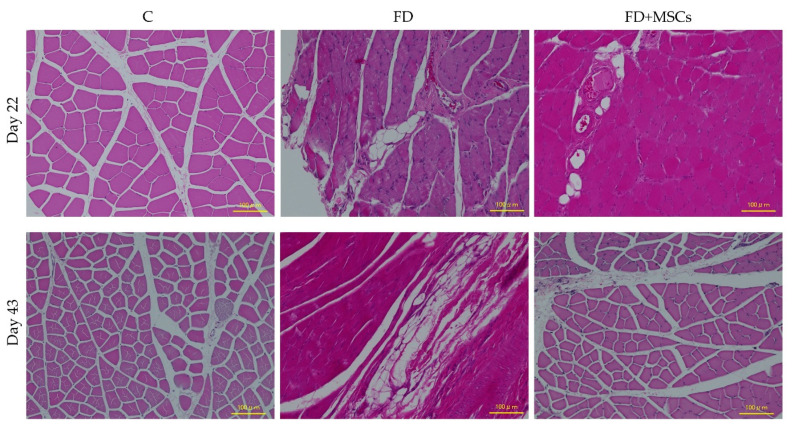
Representative histopathological images of the gastrocnemius muscle on Day 22 and Day 43 showing fatty degeneration. The images are stained with hematoxylin and eosin at a magnification of ×200. The scale bar represents 100 µm. C, control group; FD, fatty muscle degeneration group; FD+MSCs, fatty muscle degeneration with mesenchymal stem cell injection group.

**Figure 5:**
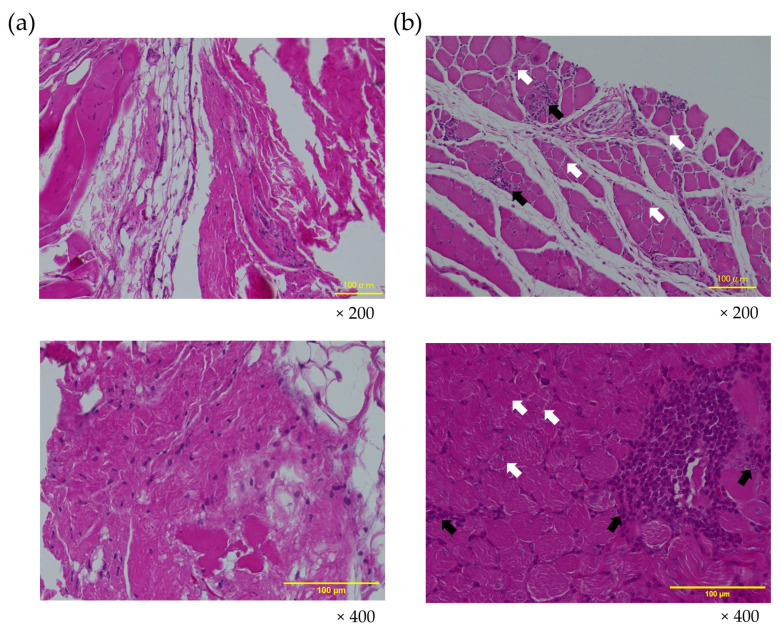
Representative histopathological changes in the gastrocnemius muscle on Day 22. (**a**) Fatty degenerated muscle in Group FD and (**b**) regenerating muscle fibers in Group FD+MSCs, stained with hematoxylin and eosin at a magnification of ×200 (upper row) and ×400 (lower row). Black arrows indicate the infiltration of inflammatory or mononucleated cells around the muscle fibers, while white arrows indicate the regeneration and formation of immature muscle fibers with central nuclei. The scale bar represents 100 µm.

Histopathological findings of the gastrocnemius muscles stained with HE on Day 22 and Day 43 are illustrated in Figure 4 and Figure 5. We did not observe any noticeable irregularities in the muscle’s healing or morphology after the administration of ADP MSCs. In Group C, no cell degeneration or fat infiltration was observed on Day 22 or Day 43.

Group FD exhibited atrophic changes, fat infiltration, and degeneration of muscle fibers on Day 22 (Figure 5a). However, in Group FD+MSCs, the regeneration of muscle fibers was evident: immature muscle fiber formation was observed, characterized by central nucleus myofiber development, and the infiltration of inflammatory cells participating in phagocytosis to remove damaged cells. The central nucleus myofibers, indicating ongoing regeneration, were sparsely observed in FD+MSCs on Day 43. Mononuclear cells (purple dots), such as inflammatory infiltrates, were observed around the endomysium in Group FD+MSCs (Figure 5b). On Day 43, fatty degeneration persisted in Group FD, whereas muscle atrophy and fat infiltration had almost recovered to control levels in Group FD+MSCs (Figure 4).

The cross-sectional area, Feret diameter, and minimal Feret diameter of muscle fibers on Day 22 and Day 43 are shown in Figure 3b. On Day 22, the cross-sectional area, Feret diameter, and minimal Feret diameter of the gastrocnemius muscle were lower in Group FD (*p* = 0.0029, *p* = 0.0029, and *p* = 0.0043, respectively) and Group FD+MSCs (*p* = 0.0331, *p* = 0.0331, and *p* = 0.0242, respectively) than in Group C. However, on Day 43, only Group FD had a lower cross-sectional area, Feret diameter, and minimal Feret diameter than Group C (*p* = 0.0004, *p* = 0.0004, and *p* = 0.0005, respectively).

### 3.2. Gene Expression Analysis by RT-qPCR

#### 3.2.1. Muscle Atrophy Marker Gene Expression

**Figure 6:**
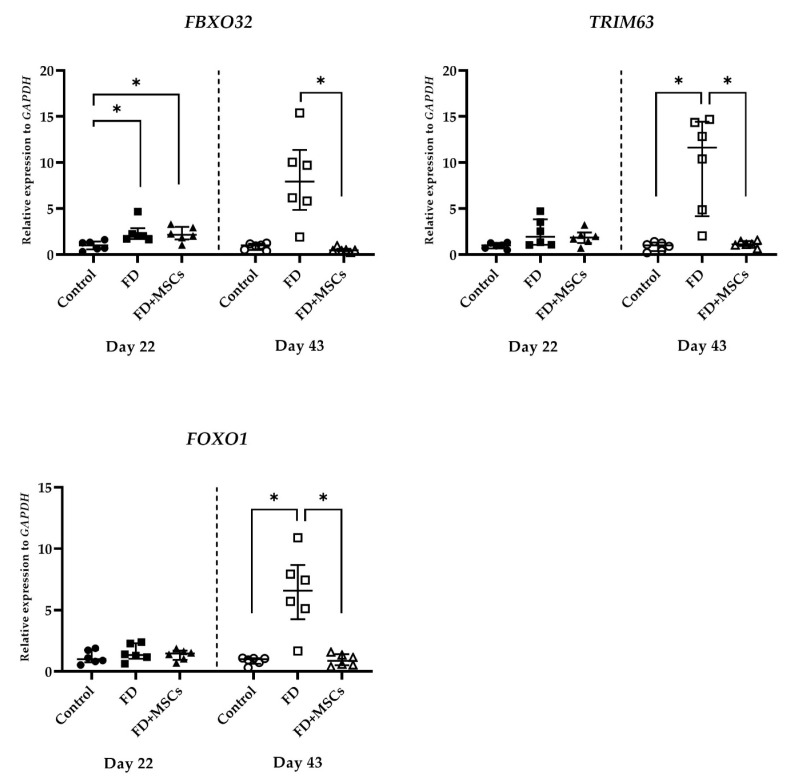
Expression of muscle atrophy marker genes in muscle tissue. RT-qPCR analysis of muscle atrophy marker gene (*FBXO32*, *TRIM63,* and *FOXO1*) expressions in the muscle harvested on Day 22 and Day 43. Data of the relative expression to *GAPDH* were analyzed with the Kruskal–Wallis test and Dunn’s multiple comparison test and shown as the median (wide horizontal line) with interquartile range (narrow horizontal line) (*n* = 6) (* *p* < 0.05). Group C on Day 22 is represented by a filled circle; Group FD on Day 22, a filled rectangle; Group FD+MSCs on Day 22, a filled triangle; Group C on Day 43, an unfilled circle; Group FD on Day 43, an unfilled rectangle; Group FD+MSCs on Day 43, an unfilled triangle; C, control; FD, fatty muscle degeneration; FD+MSCs, fatty muscle degeneration with mesenchymal stem cell injection.

The data of the muscle atrophy marker gene (*FBXO32*, *TRIM63*, and *FOXO1*) expressions, detected on Day 22 and Day 43, are shown in Figure 6. On Day 22, the expression level of *FBXO32* in Groups FD and FD+MSCs was significantly higher than that of Group C (*p* = 0.0206 and *p* = 0.0242, respectively). On Day 43, the *FBXO32* expression in Group FD was higher than that of Group FD+MSCs (*p* = 0.0013). Furthermore, *TRIM63* and *FOXO1* expressions in Group FD were higher than those in Group C (*p* = 0.0029 and *p* = 0.0062, respectively) and Group FD+MSCs (*p* = 0.0331 and *p* = 0.0175, respectively).

#### 3.2.2. Adipocyte and FAPs Marker Gene Expression

**Figure 7:**
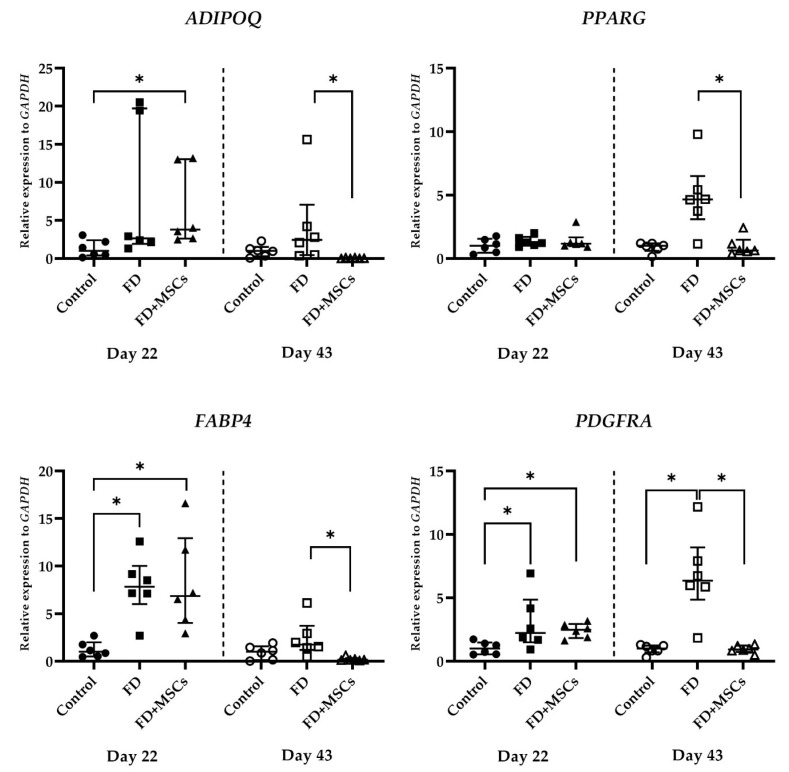
Expression of adipocyte and FAPs cell marker genes in muscle tissue. RT-qPCR analysis of adipocyte and FAPs cell marker gene (*PPARG*, *FABP4*, *ADIPOQ*, and *PDGFRA*) expressions in muscle harvested on Day 22 and Day 43. Data of the relative expression to *GAPDH* were analyzed with the Kruskal–Wallis test and Dunn’s multiple comparison test and shown as the median (wide horizontal line) with interquartile range (narrow horizontal line) (*n* = 6) (* *p* < 0.05). Group C on Day 22, filled circle; Group FD on Day 22, filled rectangle; Group FD+MSCs on Day 22, filled triangle; Group C on Day 43, unfilled circle; Group FD on Day 43, unfilled rectangle; Group FD+MSCs on Day 43, unfilled triangle; FAPs, fibro-adipogenic progenitors; C, control; FD, fatty muscle degeneration; FD+MSCs, fatty muscle degeneration with mesenchymal stem cell injection.

Adipocyte and FAPs marker gene (*ADIPOQ*, *PPARG*, *FABP4*, and *PDGFRA*) expressions, detected on Day 22 and Day 43, are shown in Figure 7. On Day 22, *ADIPOQ* expression was significantly higher in Group FD+MSCs than in Group C (*p* = 0.0242). However, on Day 43, *ADIPOQ* expression was higher in Group FD than in Group FD+MSCs (*p* = 0.0242). *PPARG* expression was higher in Group FD than in Group FD+MSCs on Day 43 (*p* = 0.0148). *FABP4* and *PDGFRA* expressions on Day 22 were higher in Groups FD and FD+MSCs than in Group C (*p* = 0.0088 and *p* = 0.0125 for *FABP4*, and *p* = 0.0449 and *p* = 0.0283 for *PDGFRA*, respectively). On Day 43, *FABP4* expression was higher in Group FD (*p* = 0.0088) than in Group FD+MSCs, and *PDGFRA* expression was higher in Group FD than in Group C (*p* = 0.0088) and Group FD+MSCs (*p* = 0.0125).

#### 3.2.3. Myogenic and Satellite Cell Marker Gene Expression

**Figure 8:**
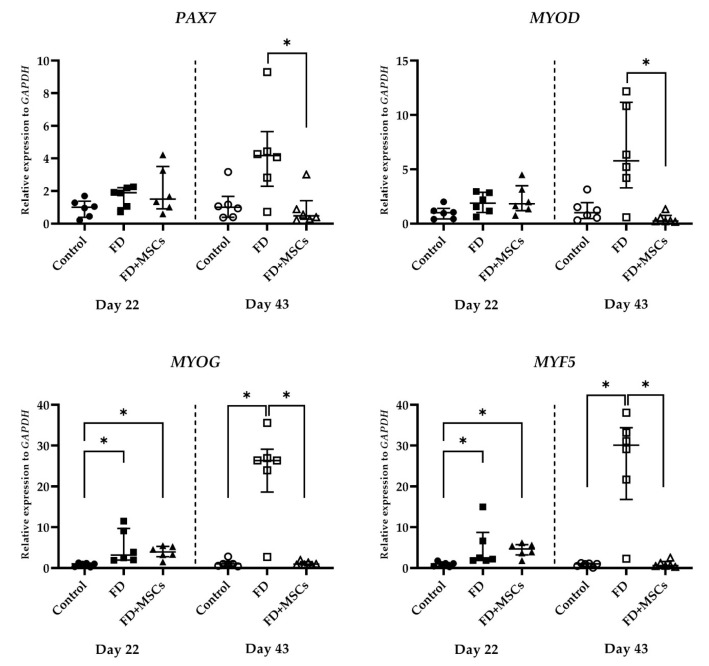
Expression of myogenic and satellite cell marker genes in muscle tissue. RT-qPCR analysis of myogenic and satellite cell marker gene (*PAX7*, *MYOD*, *MYOG,* and *MYF5*) expressions in muscle harvested on Day 22 and Day 43. Data of the relative expression to *GAPDH* were analyzed with the Kruskal–Wallis test and Dunn’s multiple comparison test and shown as the median (wide horizontal line) with interquartile range (narrow horizontal line) (*n* = 6) (* *p* < 0.05). Group C of Day 22 is represented by a filled circle; Group FD of Day 22, a filled rectangle; Group FD+MSCs of Day 22, a filled triangle; Group C of Day 43, an unfilled circle; Group FD of Day 43, an unfilled rectangle; Group FD+MSCs of Day 43, unfilled triangle; C, control; FD, Fatty muscle degeneration; FD+MSCs, fatty muscle degeneration with mesenchymal stem cell injection.

The expressions of myogenic and satellite cell marker genes (*PAX7*, *MYOD*, *MYOG*, and *MYF5*), detected on Day 22 and Day 43, are shown in Figure 8. *PAX7* and *MYOD* expressions were higher in Group FD than in Group FD+MSCs on Day 43 (*p* = 0.0206 and *p* = 0.0035, respectively). On Day 22, *MYOG* and *MYF5* expressions were higher in Group FD (*p* = 0.0105 and *p* = 0.0074, respectively) and Group FD+MSCs (*p* = 0.0105 and *p* = 0.0074, respectively) than in Group C. However, on Day 43, their expressions were higher in Group FD than in Group C (*p* = 0.0105 and *p* = 0.0125, respectively) and Group FD+MSCs (*p* = 0.0175 and *p* = 0.0148, respectively).

#### 3.2.4. Immunomodulatory Marker Gene Expression

**Figure 9:**
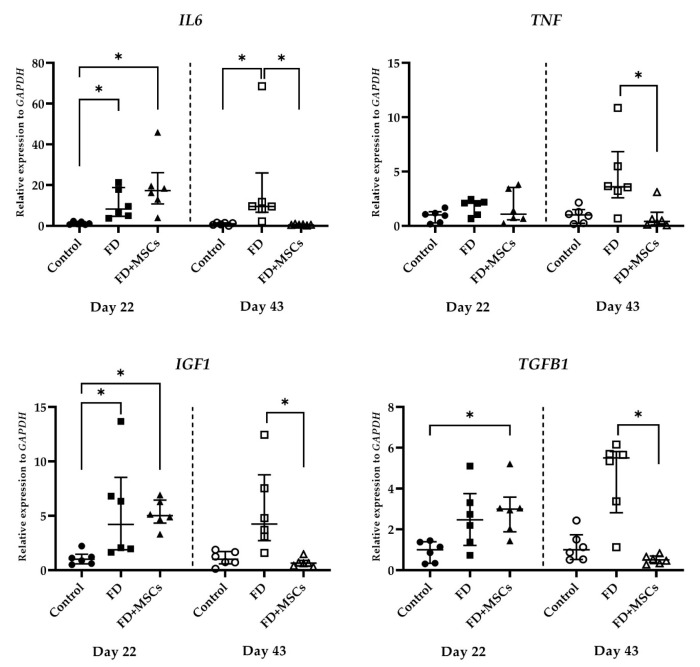
Expression of immunomodulatory genes in muscle tissue. RT-qPCR analysis of immunomodulatory marker gene (*IL6*, *TNF*, *IGF1*, and *TGFB1*) expressions in muscle harvested on Day 22 and Day 43. Data of the relative expression to *GAPDH* were analyzed with the Kruskal–Wallis test and Dunn’s multiple comparison test and shown as the median (wide horizontal line) with interquartile range (narrow horizontal line) (*n* = 6) (* *p* < 0.05). Group C on Day 22 is represented by a filled circle; Group FD on Day 22, a filled rectangle; Group FD+MSCs on Day 22, a filled triangle; Group C on Day 43, an unfilled circle; Group FD on Day 43, an unfilled rectangle; Group FD+MSCs on Day 43, an unfilled triangle; C, control; FD, fatty muscle degeneration; FD+MSCs, fatty muscle degeneration with mesenchymal stem cell injection.

Immunomodulatory marker gene (*IL6*, *TNF*, *IGF1*, and *TGFB1*) expressions, detected on Day 22 and Day 43, are shown in Figure 9. On Day 22, *IL6* and *IGF1* expressions were higher in Group FD (*p* = 0.0331 and *p* = 0.0386, respectively) and Group FD+MSCs (*p* = 0.0029 and *p* = 0.0125, respectively) than in Group C. However, on Day 43, *IL6* expression was higher in Group FD than in Group C (*p* = 0.0386) and Group FD+MSCs (*p* = 0.0024), and *IGF1* expression was higher in Group FD than in Group FD+MSCs (*p* = 0.0029). *TNF* expression was higher in Group FD on Day 43 than in Group FD+MSCs (*p* = 0.0105). *TGFB1* expression was higher (*p* = 0.0206) in Group FD+MSCs than in Group C on Day 22. However, it was higher in Group FD than in Group FD+MSCs on Day 43 (*p* = 0.0016).

## 4. Discussion

In this study, the injection of ADP MSCs reduced muscle atrophy by down-regulating the expression of muscle atrophy marker genes (*FBXO32*, *TRIM63*, and *FOXO1*) and immunomodulatory marker genes (*IL6*, *TNF*, *IGF1*, and *TGFB1*). Additionally, adipocyte infiltration and the expression of adipocyte and FAP cell marker genes (*ADIPOQ*, *PPARG*, *FABP4*, and *PDGFRA*) were suppressed, thereby reducing the progression of adipose muscle degeneration. ADP MSCs were suggested to induce muscle regeneration. This evidence indicates a therapeutic effect of ADP MSCs on fatty muscle degeneration.

This study used the sciatic nerve crush model to induce fatty muscle degeneration in the gastrocnemius muscle. The reason for this is that it is a well-established research model for neuromuscular disease and is suitable for this study. In this study, the effect of MSCs on suppressing muscle atrophy was confirmed. Muscle weight and muscle fiber size decreased by Day 22 in Groups FD and FD+MSCs. On Day 43, these measurements remained depressed in Group FD but improved in Group FD+MSCs. An et al. [33] reported that even after 3 weeks of administration of umbilical cord-derived MSCs to a mouse model of arthritis and skeletal muscle cachexia, the gastrocnemius muscle weight was reduced and the cross-sectional area of the muscle fibers showed no improvement. Dosage and frequency of administration have been pointed out as the cause. Song et al. [34] reported an increase in muscle mass and cross-sectional area of muscle fibers in atrophic muscles after 8 weeks of treatment with umbilical cord-derived MSCs in mice. Thus, our results suggest that ADP MSCs promoted muscle regeneration by Day 43.

During muscle atrophy, the muscle atrophy marker genes *TRIM63* and *FBXO32* are upregulated [35]. *FOXO1* is a transcription factor and the *FOXO* family plays a crucial role in regulating the expression of the *MuRF-1* (*TRIM63*) and *atrogin-1* (*FBXO32*) genes [36,37]. In the present study, the expression of these muscle atrophy marker genes increased in Group FD. In contrast, in Group FD+MSCs, *FBXO32* expression increased only by Day 22. This is consistent with reports that umbilical cord-derived MSCs alleviate muscle atrophy by reducing *atrogin-1* (*FBXO32*) and *MURF-1* (*TRIM63*) expression [34,38].

Fatty muscle degeneration in the gastrocnemius muscle was induced by crushing the sciatic nerve. Fat infiltration was observed in the HE-stained sections on Day 22. This was similar to the findings of Shinohara et al. [30] who reported muscle atrophy and fatty infiltration in the gastrocnemius muscle was observed in the nerve-crushed group after 4 weeks. *ADIPOQ* is a lipid metabolism-related gene [39], functions as an adipokine, and is also expressed in skeletal muscle [40]. The expression of *ADIPOQ* was upregulated during the regeneration process of unloading-associated atrophied soleus muscle [41]. *FABP4* is an adipocyte gene essential for fatty acid transport and fat deposition [39]. *PDGFRA* gene is a marker of FAP cells, also known as mesenchymal progenitor cells [42], and is a major factor in fatty infiltration in skeletal muscle degeneration [43]. FAPs play an important role in muscle regeneration, and dysregulation of FAPs has been described to cause fatty infiltration, muscle atrophy, and impaired muscle regeneration [3]. In the present study, the expression of adipocyte differentiation markers increased on Day 22 in Groups FD and FD+MSCs, suggesting ectopic fat deposition due to the action of FAPs. However, on Day 43, their expression was lower in Group FD+MSCs than in Group C. Studies in sheep [28] and rabbits [44] using rotator cuff tear models showed a reduction in fat infiltration after ADP MSC transplantation. In sheep rotator cuff tear muscle, atrophy of fatty degenerated muscle was inhibited after 6 weeks of MSC administration [26]. To the best of our knowledge, this study is the first to demonstrate that ADP MSCs reduce the expression of adipogenic and FAP genes and mitigate fatty infiltration in the gastrocnemius muscle of rats.

Satellite cells are muscle stem cells that reside beneath the basal lamina surrounding each myofiber. They are responsible for skeletal muscle repair mechanisms by differentiating into myoblasts during regeneration [45]. The muscle microenvironment plays an important role in the myogenic differentiation of MSCs and in regulating the immune system regulation through cytokine signal transmission [46]. They express the transcription factor *PAX7* in a resting state and co-express *PAX7* and *MYOD* when activated [47]. *MYOD* and *MYOG* are early myogenic markers [48]. *MYF5* is expressed in the early stages prior to terminal myogenesis and subsequently acts as an important myogenic factor in skeletal muscle [49]. The increased expression levels of *MYOG* and *MYF5* in Groups FD and FD+MSCs on Day 22 of this study suggest the initiation of the regeneration and early differentiation stage of myoblasts. In Group FD+MSC, phagocytosis, inflammatory cell infiltration, and formation of regenerating central nucleus myofibers were observed on Day 22, suggesting early muscle regeneration and muscle repair effects by ADP MSCs. On Day 43, the central nucleus myofibers were sparsely observed in FD+MSCs. This could be due to their higher occurrence during the initial stages of muscle repair, gradually decreasing as the muscle fibers mature. The high expression of *PAX7*, *MYOD*, *MYOG*, and *MYF5* in Group FD on Day 43 could be interpreted as the myofibers still functioning in the spontaneous reparative stage and attempting to replace damaged muscle fibers. Persistent *PAX7* expression may indicate proliferation and delayed onset of differentiation [47], as downregulation of *PAX7* occurs when myogenic differentiation begins. In Group FD+MSCs, the expression of these markers was lower on Day 43 than in Group FD, suggesting that the ability to regenerate muscle was achieved by the administration of ADP MSCs. This may be explained by the activating effect of MSCs on satellite cells for myogenesis and muscle protein synthesis [22].

Increased inflammatory markers are associated with reduced muscle mass [50] and reduced mobility function [51,52,53]. Persistent and excessive inflammation inhibits the repair of damaged tissue and induces muscle atrophy [54]. Increased inflammatory cytokines lead to persistent inflammation. *IL6* and *TNFα* are proinflammatory cytokines secreted by adipocytes. Their concentration correlates with the percentage and distribution of fat tissue in the body [55]. The expression of *IL6* and *TNF* increased in a rat model of muscle fatty degeneration induced by nerve crushing [30]. The regenerative process was then completed as inflammatory markers decreased [56]. An et al. [33] reported that the levels of inflammatory cytokines such as *TNFα* and *IL6* were higher 3 weeks after injection of human umbilical cord-derived MSCs compared to the collagen-induced arthritis group. In the present study, we found that *IL6* was highly expressed on Day 22 in Group FD+MSCs but was no different than in Group FD. Therefore, we concluded that this finding was not due to the administration of ADP MSCs. The expression of inflammatory markers was higher in Group FD than in Group FD+MSCs on Day 43. This suggests that inflammation persisted in Group FD, while ADP MSCs reduced inflammation in Group FD+MSCs.

*IGFs* are two small polypeptides (~7 kDa) that regulate stem cell survival, self-renewal, and differentiation, and their expression is related to FGF [57]. *IGF1* is produced in the liver as well as the heart, lungs, muscles, testes, stomach, kidneys, and brain [58]. Increased expression of *IGF1* stimulates MSCs through a paracrine effect on muscle progenitor cells, enhancing their healing ability [21]. This also induces the initial stages of skeletal muscle regeneration via satellite cell activation [59,60]. *IGF1* expression was higher in Group FD than Group FD+MSCs on Day 43, suggesting that muscle regeneration was still ongoing.

*TGFB1* in rat skeletal muscles is upregulated in response to damage and denervation [61]. *TGFB1* is a potent inhibitor of myoblast differentiation and is involved in skeletal muscle atrophy and connective tissue formation [62]. Umbilical cord-derived MSCs have been reported to prevent muscle atrophy induced by *TGFB1* [38,63]. Neutralization of *TGFB1* resulted in increased myoblast proliferation and differentiation in vitro [61]. *TGFB1* in Group FD+MSCs was higher than Group C on Day 22, but lower than Group FD on Day 43, suggesting that MSCs suppressed muscle atrophy. Although *TGFB1* promotes inflammation [64], Liu et al. reported that *TGFB* secreted by MSCs suppresses the inflammatory reactions [65]. Further clarification of the origin of *TGFB* is needed to determine whether *TGFB* is secreted by MSCs or by local tissues.

The study has several limitations. First, the dose, frequency, and route of administration of ADP MSCs were not varied. Furthermore, the viability and longevity of cells injected into muscle have not been evaluated. The efficacy of transplanted MSCs may diminish over time due to cell proliferation and differentiation. Future studies should confirm the dose-dependent effect, the long-term sustainability of the treatment, potential side effects after transplantation, and methods for identifying MSCs after transplantation. Second, although MSCs may exhibit various regenerative effects depending on their origin, this study used MSCs from only one source (derived from adipose tissue) and did not evaluate tissue-specific differences. Third, we observed the infiltration of inflammatory cells in fatty degenerated muscle treated with MSCs but did not quantify mononuclear cells. Inflammation plays a crucial role in regulating skeletal muscle regeneration [66]. In the future, we aim to clarify the difference and relationship between inflammation and muscle regeneration. Fourth, we could not clarify whether the regenerative effects of MSCs are due to myogenic or induced effects on endogenous muscle stem cells, or both. It was a limitation of this study that we could not directly provide evidence of MSC involvement in the observed changes. However, as the initial step, we explored the genetic markers involved in the regeneration process of fatty muscle degeneration after the injection of MSCs. Based on these results, the regenerative mechanisms and molecular pathways of MSCs should be clarified through genetic manipulations and/or secretome analysis of each marker. Fifth, this study primarily focused on molecular analysis. Functional testing with muscle repair was not performed. Future studies should include these tests, such as assessments of muscle strength and endurance, for a more comprehensive understanding. Additionally, clinically applicable diagnostic methods, such as PCR testing with muscle biopsy, need to be developed to evaluate the prognostic value of MSC treatment in fatty muscle degeneration. Finally, it is necessary to clarify whether the cytokines involved in muscle regeneration originate from MSCs or from local secretion, as well as their interactions.

## 5. Conclusions

This study demonstrated the effectiveness of ADP MSCs administration in inhibiting both fatty degeneration and atrophy in the gastrocnemius muscle of rats. Muscle regeneration by ADP MSCs was achieved by Day 43, with the formation of immature myofibers observed on Day 22. ADP MSCs inhibited the progression of fatty muscle degeneration by suppressing the expression of muscle atrophy marker genes (*FBXO32*, *TRIM63*, and *FOXO1*) and marker genes associated with adipocytes and FAPs cells (*PPARG*, *FABP4*, *ADIPOQ*, and *PDGFRA*), as well as inflammation-related genes (*IL6*, *TNF*, *IGF1*, and *TGFB1*). Based on our results, ADP MSCs induce anti-inflammatory effects, inhibit fat accumulation, and promote muscle regenerative processes, suggesting that they are a promising treatment potential for fatty muscle degeneration and atrophy.

## Figures and Tables

**Table 1:** Primer sequences.

Gene Name		Direction	Primer Sequences (5′-3′)
Housekeeping gene	*GAPDH*	Forward	CCTGTTCTAGAGACAGCCGC
		Reverse	ATCCGTTCACACCGACCTTC
Immunomodulatory markers	*IL6*	Forward	CCACCCACAACAGACCAGTA
		Reverse	TCTGACAGTGCATCATCGCT
	*TNF*	Forward	TCTTCAAGGGACAAGGCTGC
		Reverse	CGGAGAGGAGGCTGACTTTC
	*TGFB1*	Forward	ATGCCAACTTCTGTCTGGGG
		Reverse	GGTTGTAGAGGGCAAGGACC
	*IGF1*	Forward	TGGTGGACGCTCTTCAGTTC
		Reverse	TCCGGAAGCAACACTCATCC
Adipocyte and FAPs markers	*PPARG*	Forward	AGCTCTGTGGACCTCTCTGT
		Reverse	GTCAGCTCTTGTGAACGGGA
	*PDGFRA*	Forward	AGTGCTTGGTCGGATCTTGG
		Reverse	GAGCATCTTCACAGCCACCT
	*FABP4*	Forward	AACTGGGCGTGGAATTCGAT
		Reverse	CACATGTACCAGGACCCCAC
	*ADIPOQ*	Forward	TAATTCAGAGCAGCCCGTAG
		Reverse	TGGGGATAACACTCAGAACC
Atrophy markers	*FBXO32*	Forward	TGCTCCGTCTCACTTTCACC
		Reverse	AGGGGCCTTCTGAAGTGTTG
	*TRIM63*	Forward	ACCAAGGAAAACAGCCACCA
		Reverse	GGATCAGGGCCTCGATGAAG
	*FOXO1*	Forward	AGCTGCATCCATGGACAACA
		Reverse	TCATCATTGCTGTGGGACCC
Myogenic and satellite cell markers	*PAX7*	Forward	CAAGATGCTGGGACACTCGT
		Reverse	ATGCCAGAGAGCCAGTTTCC
	*MYOD*	Forward	CGACTCTTCAGGCTTGGGTT
		Reverse	TGTCGCAAAGGAGCAGAGAG
	*MYOG*	Forward	GGCAATGCACTGGAGTTTGG
		Reverse	CGTAAGGGAGTGCAGGTTGT
	*MYF5*	Forward	ATGGACATGACGGACAGCTG
		Reverse	TGCGACTCTTGGCTCAAACT

## Data Availability

Data are contained within the article.
